# Development of a Cost-Effective Airborne Remote Sensing System for Coastal Monitoring

**DOI:** 10.3390/s151025366

**Published:** 2015-09-30

**Authors:** Duk-jin Kim, Jungkyo Jung, Ki-mook Kang, Seung Hee Kim, Zhen Xu, Scott Hensley, Aaron Swan, Michael Duersch

**Affiliations:** 1 School of Earth and Environmental Sciences, Seoul National University, Seoul 151-742, Korea; E-Mails: oberonia@naver.com (J.J.); mook0416@naver.com (K.-m.K.); dalcomeboy@snu.ac.kr (S.H.K.); xuzhen426@snu.ac.kr (Z.X.); 2 Jet Propulsion Laboratory, California Institute of Technology, Pasadena 91109, CA, USA; E-Mail: scott.hensley@jpl.nasa.gov; 3 IMSAR LLC, Springville 84663, UT, USA; E-Mails: aarons@imsar.com (A.S.); michaeld@imsar.com (M.D.)

**Keywords:** airborne remote sensing, synthetic aperture radar, thermal infrared, coastal monitoring, interferometry

## Abstract

Coastal lands and nearshore marine areas are productive and rapidly changing places. However, these areas face many environmental challenges related to climate change and human-induced impacts. Space-borne remote sensing systems may be restricted in monitoring these areas because of their spatial and temporal resolutions. In situ measurements are also constrained from accessing the area and obtaining wide-coverage data. In these respects, airborne remote sensing sensors could be the most appropriate tools for monitoring these coastal areas. In this study, a cost-effective airborne remote sensing system with synthetic aperture radar and thermal infrared sensors was implemented to survey coastal areas. Calibration techniques and geophysical model algorithms were developed for the airborne system to observe the topography of intertidal flats, coastal sea surface current, sea surface temperature, and submarine groundwater discharge.

## 1. Introduction

Coastal areas are continuously changing, and it is difficult to gain a systematic understanding of the coastal environment using only *in situ* surveys of narrow coastal areas. Obtaining physical information in coastal areas other than some places where observation takes place in real-time using sensors installed on buoys, light beacons, or tidal stations is often not possible. In particular, almost no high-resolution, two-dimensional data are being gathered from coastal areas with many islands and where there is a large tidal range with intricate shorelines. Of course, out at sea (at least 25 km from the coastline), wind speed/direction and geostrophic current are estimated through man-made satellites equipped with scatterometers (e.g., QuikScat and NSCAT) or altimeters (e.g., JASON-1, TOPEX-Poseidon, and ENVISAT RA-2). Sea surface temperature (SST) is also being calculated with satellites such as MODIS and NOAA. However, these man-made satellites cannot provide precise quantitative information about coastal waters because their spatial resolution is approximately 25 km (approximately 1 km for NOAA sea surface temperature). On the other hand, high-density digital elevation models (DEMs) of the surface terrain of inland areas are also available from shuttle radar topography mission (SRTM) or recently, TanDEM-X mission. However, it is not easy to monitor deposition and erosion along coastal areas using these data because valid DEMs or differential interferograms cannot be generated under the changing tide and low coherence conditions of coastal areas (in particular, intertidal flats).

Synthetic aperture radar (SAR) sensors are not only able to acquire high-resolution images (<5 m) but, in contrast to optical sensors, they can also observe under any conditions, whether day or night, and regardless of weather conditions. SAR sensors even provide continuous data during extended cloudy weather. Notably, when serious coastal disasters such as oil pollution occur, these sensors offer data immediately regardless of weather conditions, making them a useful tool for minimizing damage [1]. At the same time, infrared sensors can provide data outside the visible band of light, meaning more than what the human eye can see. In particular, thermal infrared sensors for wavelengths at approximately 10 μm, which are useful for measuring the temperature of objects, observe even at night without sunlight and are well-suited for analyzing the characteristics of objects. However, when fitted to man-made satellites, these bulky thermal infrared sensors are limited in their ability to obtain high-resolution images. Because satellite observational windows are constrained by their orbital geometry relative to the earth, their viewing opportunities are limited both temporally and in aspect. However, if these sensors are mounted on an aircraft, then it is easy to select the desired perspectives and areas, making it possible to obtain high-resolution data more quickly.

The availability of data on coastal sea surface temperature is important in fish farm management and in various other fields that influence the land, such as the areas of river and groundwater discharge. Likewise, coastal sea surface current is useful for the management of vessels and leisure lifestyle-related activities. In addition, high-precision topography information on intertidal flats is useful for monitoring the processes of deposition and erosion over time. However, SST and altimetry data obtained using man-made satellites for coastal areas with many islands is not precise enough because of their relatively low-resolution (~25 km) imaging capabilities. Many floating objects in coastal seas are also factors in degrading the quality of SST and altimeter data [2]. For these reasons, it is nearly impossible to obtain accurate and high-resolution two-dimensional imaging data on sea surface temperature and current in intricate coastal areas with many islands. In addition, DEMs being used globally cannot currently provide useful topography information in coastal areas.

In coastal areas, some thermal anomalies such as groundwater plume discharge, seawater heating due to tidal flat, and local upwelling, can be as small as less than 5 m. Sea surface velocities along small tidal channels are also important parameter to be monitored in coastal areas. Small scale of cliff collapse and coastal erosion is often occurred here and there. All these coastal phenomena have relatively small-scales and have been little monitored using space-borne satellite systems. Thus, the purpose of this paper is to describe an airborne remote sensing system that can obtain high-resolution data (<0.5 m) on sea surface temperature, sea surface current, and the topography of coastal areas that closely affect human life. In particular, low-cost sensors that are readily available and can be easily purchased are assembled in this airborne remote sensing system. The study also includes the development of test flights, calibration, and parameter extraction algorithms in order to achieve adequately high-precision data for coastal area monitoring.

## 2. Configuration of the Airborne Remote Sensing Sensors

### 2.1. Synthetic Aperture Radar (SAR)

To generate two-dimensional images, SAR sensors generate their own electromagnetic waves that can radiate through a side-looking antenna. These signals are then received back after being scattered from objects. A high-resolution SAR image can be obtained in the range direction (perpendicular to the flight direction) and azimuth direction (parallel to the flight direction) by chirp-pulse compression of returning signals recorded in time order and by the compression of Doppler history caused by sensor’s position changes, respectively. Based on the frequency of the electromagnetic waves emitted by the SAR antenna, the mostly used frequencies for global remote sensing include the L-band (~1.2 GHz), C-band (~5.3 GHz) and X-band (~9.5 GHz). Among these, the X-band has a relatively shorter wavelength and reacts sensitively to capillary waves on the sea surface. For this reason, the X-band is commonly used to analyze physical oceanic phenomena that affect these surface capillary waves, such as sea surface wind, wave, and current [3]. In addition, with the limitations imposed by fitting on a small aircraft, more precise topography measurements can be obtained at X-band because of the better baseline to wavelength ratio that provides increased sensitivity to height.

**Figure 1:**
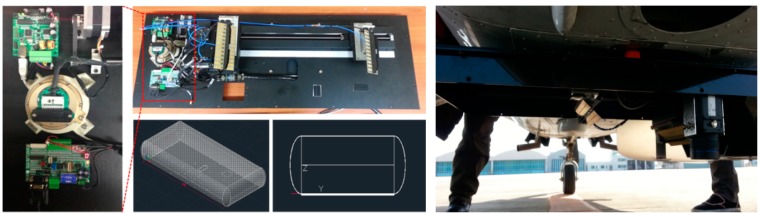
Airborne SAR and TIR systems. (**Left**): Mounting frame for adjusting baseline of SAR antennas; (**Right**): Picture of SAR and TIR systems as mounted on the belly of the aircraft.

In this study, we attempted to build an SAR system using a X-band wavelength, and thus we adopted an RF device called a NanoSAR from ImSAR LLC (Springville, UT, USA, Figure 1). This device (dimensions: 16 cm horizontal, 19 cm depth and 11.5 cm height) weighs a total of approximately 1.6 kg. Thus, it can be easily installed on any aircraft. The SAR signal can be emitted from a patch-type antenna with a central frequency of 10.25 GHz (X-band) and maximum signal bandwidth of 500 MHz. Ground resolution is adjustable between 0.3 m and 2 m. Swath width varies depending on the ground resolution (*i.e.*, 600 m for 0.3 m resolution and 4 km for 2 m resolution) and the number of channels (*i.e.*, the swath width will be half if dual-channel mode is used). Swath sizing is scales in this manner because the radar digital system records a fixed number of samples per pulse (Table 1).

To calculate ground topography and measure the speed of moving objects, we constructed a mounting system that is possible to work for interferometric mode. A patch antenna with the same performance specifications was additionally installed for the dual-channel SAR system. The first antenna was set to be able to both send and receive SAR signals, and the second antenna was made to receive only the SAR signals sent from the first antenna. In addition, the distance between the two antennas (baseline) was designed to be adjustable between 10 and 60 cm in order to acquire optimal data depending on the topographical gradient of the terrain and on the sensory speed range of moving objects, (Figure 1). This makes it possible to extract topographic height (DEM: Digital elevation model) by using two antennas arranged perpendicularly to the aircraft traveling direction (XTI: Cross-track interferometry), and to measure the speed of moving objects from the two antennas arranged front-to-back in the aircraft travelling direction (ATI: Along-track interferometry). This was connected to a dual-frequency global positioning system (GPS) antenna and an inertial measurement unit (IMU) device to acquire positional and attitudinal information needed for geometric correction of the SAR data.

**Table 1:** Specification of the NanoSAR sensor.

Parameter	Value
Frequency	X-band (10.25 GHz)
Bandwidth	500 MHz
Range resolution	0.3, 0.5, 1, 2 m
Swath width	300 m–4 km
Operating mode	StripMap, (Spotlight)
Supply voltage	12–18 V

### 2.2. Thermal Infrared (TIR)

In this study, an A615 model device from FLIR Systems Inc. (Wilsonville, OR, USA) was utilized for the airborne sensor to measure the temperature of the Earth’s surface. Detailed specifications of this model are listed in Table 2. The device was originally developed to measure the temperature of nearby objects, but it can be adequately utilized for airborne remote sensing if appropriately calibrated. First, in order to geometrically correct the measured thermal infrared data, a GPS and gyro sensor (MTi-G of Xsens Co., Enschede, Netherlands) were attached to the A615 device, with a SE-342 device to measure temperature and humidity for radiometric correction. After using a focal length look-up table (which focal length value should be set depending on the aircraft altitude) from the laboratory experiments, the system was configured so that the focal length could be adjusted based on GPS-provided altitude data. The mounting system between the sensor and aircraft was built as shown on the right side of Figure 1.

**Table 2:** Specifications of the FLIR A615 thermal infrared sensor.

Parameter	Value
Field of view (FOV)	45° × 34° (55° diagonal)
Spatial resolution (IFOV)	1.23 mrad
Spectral range	7.5–13 μm
Focal length	13.1 mm
Thermal sensitivity	<0.05 °C at 30 °C
IR resolution	640 × 480 pixels

### 2.3. Configuration of Remote Sensing Sensors on Aircraft

Cessna 206 or 208 were used as a platform of the remote sensing systems. Both aircraft models were modified to have a round hole in the bottom of the fuselage. The SAR and TIR sensors were installed on a rectangle plate frame (1000 mm × 420 mm) and this frame was attached to a connector mounted to the inside of the aircraft through the round hole (Figure 1), with a shock-absorbing spring providing protection from shocks during takeoff and landing. Electric powers for SAR and TIR sensors were provided from batteries (MaxPower300, SPS Inc., Daejeon, Korea) located inside of aircraft. Command and control were carried out by on-boarding workstations (laptop computers) for each sensor through multiple cables of 37 pins, RS-232, and RJ45 jack for 10/100 Ethernet. Because the Cessna 206 or 208 aircraft were modified to have a round hole in the bottom of the fuselage, limited persons can be boarded on the airplane. In order to maintain stable path and motion, flight is carefully controlled using a Garmin 795 navigation system.

## 3. Calibration of Airborne Sensors

### 3.1. Radiometric and Interferometric Calibration of SAR Sensor

**Figure 2:**
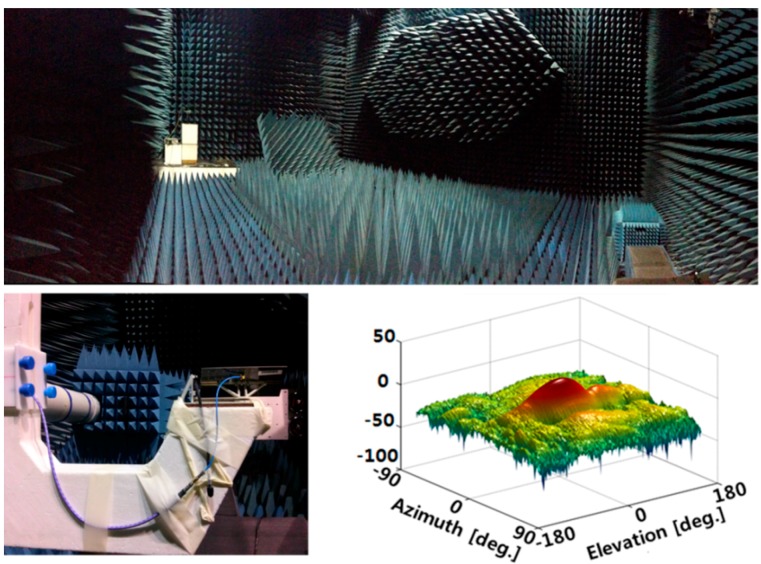
Three-dimensional antenna pattern measurements in an anechoic chamber.

**Figure 3:**
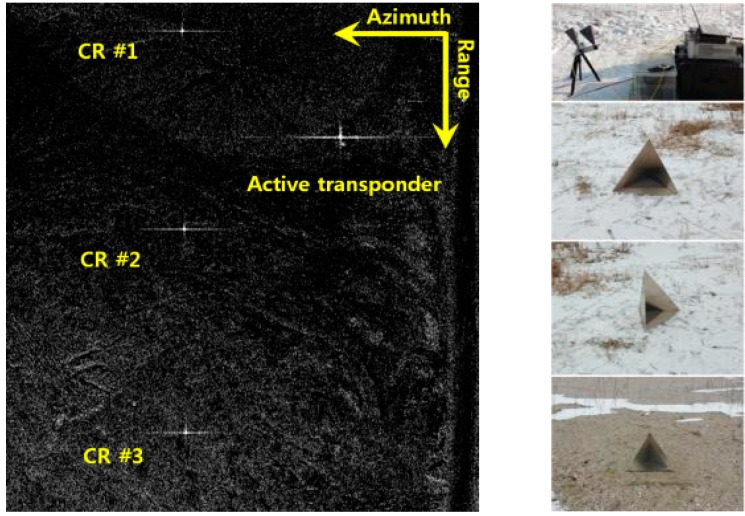
(**Left**): SAR image of the calibration site; (**Right**): Photos of the active transponder and corner reflectors installed at the calibration site (Shiwha, Hwasung-Si).

To obtain quantitative information (the radar backscattering coefficient) from SAR data, SAR sensors must be radiometrically calibrated. For general SAR calibration, the patch antenna pattern both in elevation and azimuth directions, as well as the calibration constant for the entire system, must be known precisely. In this study, the airborne SAR’s antenna pattern was checked in the laboratory (anechoic chamber) through 3-D antenna pattern measurement (Figure 2). Then the overall calibration constant (73.3 dB) was obtained by measuring the difference between the SAR observed RCSs for corner reflectors, which were arrayed along the range direction in the area of Shihwa tideland in Hwaseong, Gyeonggi-Do, and the theoretical RCS values (Figure 3).

Proper interferometric calibration of the SAR system is essential to obtaining accurate DEMs. To measure yaw and pitch angle biases of IMU on-boarded to aircraft, the Doppler centroid as a function of range was derived from SAR range compressed data. The expected Doppler centroid and the derivatives of the Doppler centroid were calculated using the values of yaw, pitch, and platform velocity measured from IMU and GPS. By comparing the measured and expected Doppler centroids, the angle biases were estimated. For the correction of differential time delay (the time difference between channels due to different cable lengths), cross correlations between slant range images obtained from both channels were applied and the range offset was used to determine the time delay. The common range delay (the unmeasured electrical path lengths in the radar) can be solved for simultaneously with the platform position error. The error of the range to a target is given by [4]:

(1) $$\frac{\partial \rho }{\partial P_{s}}\text{Δ}P_{s}+\frac{\partial \rho }{\partial P_{c}}\text{Δ}P_{c}+\frac{\partial \rho }{\partial P_{h}}\text{Δ}P_{h}+\frac{\partial \rho }{\partial \tau }\text{Δ}\tau =\text{Δ}\rho$$

where, $\frac{\partial \rho }{\partial P_{s}}=\cos\beta$, $\frac{\partial \rho }{\partial P_{c}}=\sqrt{1-{\left(\frac{\cos\beta }{\sin\theta }\right)}^{2}}\sin\theta$, $\frac{\partial \rho }{\partial P_{h}}=-\cos\theta$, $\frac{\partial \rho }{\partial \tau }=1$, β is the angle between the aircraft velocity and the look direction, θ is the look angle, Δτ is the common range delay, $P_{s}$, $P_{c}$, and $P_{h}$ are the platform position components in along track, across track, and vertical directions, respectively. The slant range location errors of each corner reflector (Figure 3) were measured and the Equation (1) was solved using singular value decomposition to determine the platform position errors and common range delay.

The baseline and interferometric phase errors can be determined from the errors in the positions of each target, $\text{Δ}\vec{T}$, which can be given by [4,5]:

(2) $$\frac{\partial \vec{T}}{\partial B}\text{Δ}B+\frac{\partial \vec{T}}{\partial \alpha }\text{Δ}\alpha +\frac{\partial \vec{T}}{\partial \phi }\text{Δ}\phi =\text{Δ}\vec{T}$$

where, $\frac{\partial \vec{T}}{\partial B}=\frac{\rho }{B}\tan\left(\theta -\alpha \right)\left[\begin{array}{c} 0\\ -\cos\theta \\ -\sin\theta \end{array}\right]$, $\frac{\partial \vec{T}}{\partial \alpha }=\rho \left[\begin{array}{c} 0\\ \cos\theta \\ \sin\theta \end{array}\right]$, $\frac{\partial \vec{T}}{\partial \phi }=\left(\frac{-\lambda \rho }{2\pi B\cos\theta -\alpha }\right)\left[\begin{array}{c} 0\\ \cos\theta \\ \sin\theta \end{array}\right]$, B is baseline length, α is the baseline orientation angle, ϕ is the interferometric phase, and λ is the radar wavelength. The Equation (2) was solved using singular value decomposition to determine the error in the baseline length (ΔB), the error in the baseline roll angle (Δα), and the error in the interferometric phase (Δϕ). SAR data acquisitions with multiple passes over the calibration site (Shiwha, Hwasung-Si) were carried out and the exact locations for corner reflectors were measured using Differential Global Positioning System (DGPS) techniques.

### 3.2. Radiometric and Geometric Calibration of TIR Sensor

**Figure 4:**
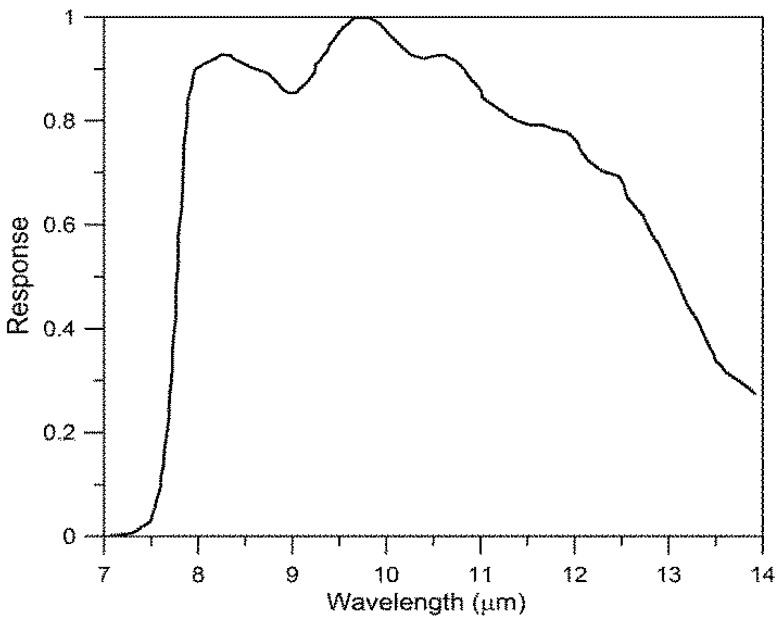
Spectral response curve for FLIR A615.

**Figure 5:**
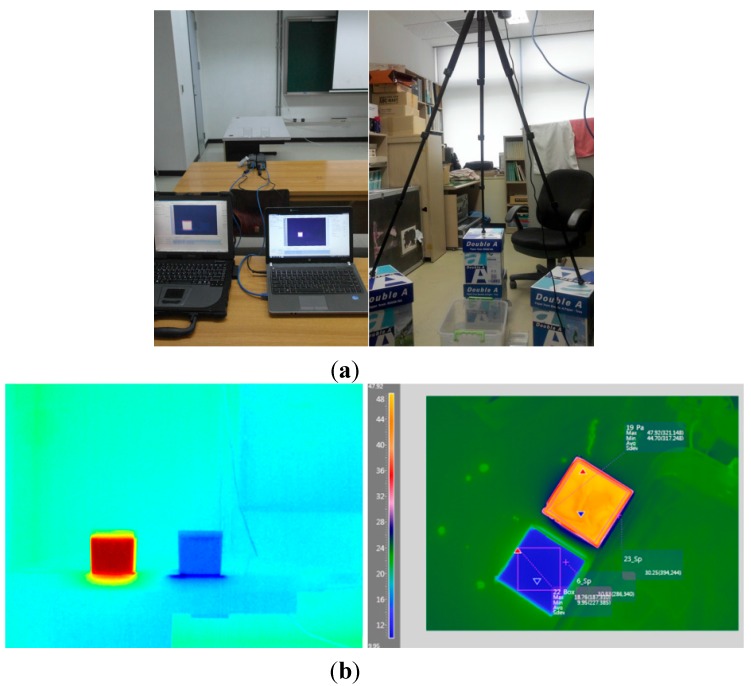
(**a**) Photos of laboratory experiments and (**b**) TIR images.

To quantitatively measure sea surface temperature using the TIR sensor, atmospheric corrections between the ocean surface and the airborne sensor were performed. The radiant energy entering a sensor can be corrected using a radiative transfer model that not only takes into account the radiant energy emitted by an object but also the radiant energy emitted again after having entered the object from the surrounding environment, and the energy absorbed in the atmospheric water vapor while passing through the atmosphere that is then released from the atmosphere itself to enter the sensor [6,7]. There were various radiative transfer models for thermal infrared bandwidth and this study used the Santa Barbara DISORT atmosphere radiative transfer model (SBDART) [8]. Weather research and forecast (WRF) model results of temperature, water vapor, and ozone profiles were used as model entry parameters of SBDART. Because the WRF model results may not be accurate enough to compensate for the contribution of the atmosphere, the temperature and water vapor profiles were matched to the observed values recorded in the thermometer/hygrometer data logger (SE-342) installed on the aircraft. Because the A615 thermal infrared sensor from FLIR Systems has different spectral responses (filter types) depending on the wavelength (Figure 4) and records “raw count” as the sum of the total energy entering through the wavelength band (7.5–13 μm) at each unit of time, the values of the raw count were converted into a quantity of “total power”, which has more physical meaning, using empirical relationship (Equation (3)) derived from various indoor experiments (Figure 5), and then the sea surface temperatures were calculated by the process of atmospheric correction using SBDART [9].

(3) Total power=0.0133×raw count−57.798

Ground point control (GCP) is commonly used for the geometric correction of remote sensing data. However, it is difficult to do geometric correction using GCPs for the large amount of data acquired by airborne remote sensors. Furthermore, clear surface features may not be visible in the thermal infrared images, so that it can be difficult to select GCPs. In particular, geometric correction is almost impossible with the data acquired over the sea because there are almost no recognizable geographical features. Therefore, automatic geometric correction is needed even if it may involve some positional errors. In this study, GPS and IMU sensors were installed on the aircraft to obtain position (*P_x_*, *P_y_*, *P_z_*) and attitude (yaw, pitch, roll) information. Because the MTi-G device from Xsens has both a GPS receiver and gyro sensor, it can simultaneously collect position, attitude, and time information. To correct the images acquired by TIR sensors, the acquisition time of the TIR sensor was synchronized with the time of MTi-G device. With the sensor specifications given in Table 2, such as focal length (f), instant field of view (IFOV), and field of view (FOV), the ground coordinates corresponding to the four corner points (*O_1_*, *O_2_*, *O_3_*, and *O_4_*) of the TIR images were calculated using the simple collinearity equation in Universal Transverse Mercator (UTM) coordinate system [10]:

(4) $$\begin{array}{c} \begin{array}{c} O_{1}=\left[320\times \text{f}\times {\text{IFOV}}_{1},\;240\times \text{f}\times {\text{IFOV}}_{2},\;-\text{f}\right]\\ O_{2}=\left[-320\times \text{f}\times {\text{IFOV}}_{1},\;240\times \text{f}\times {\text{IFOV}}_{2},\;-\text{f}\right] \end{array}\\ \begin{array}{c} O_{3}=\left[320\times \text{f}\times {\text{IFOV}}_{1},\;-240\times \text{f}\times {\text{IFOV}}_{2},\;-\text{f}\right]\\ O_{4}=\left[-320\times \text{f}\times {\text{IFOV}}_{1},\;-240\times \text{f}\times {\text{IFOV}}_{2},\;-\text{f}\right] \end{array} \end{array}$$

These are the image coordinates ([$O_{x},\;O_{y},\;O_{z}$]) that correspond to the four corner points of TIR images, assuming that there is no attitude change. However, if the attitude of the aircraft is varied, the four coordinates also change accordingly. This can be calculated using the following equations:

(5) $$\begin{array}{l} m_{11}=\cos\theta_{p}\cos\theta_{y}\\ m_{12}=\sin\theta_{r}\sin\theta_{p}\cos\theta_{y}+\cos\theta_{r}\sin\theta_{y}\\ m_{13}=-\cos\theta_{r}\sin\theta_{p}\cos\theta_{y}+\sin\theta_{r}\sin\theta_{y}\\ m_{21}=-\cos\theta_{p}\sin\theta_{y}\\ m_{22}=-\sin\theta_{r}\sin\theta_{p}\sin\theta_{y}+\cos\theta_{r}\cos\theta_{y}\\ m_{23}=\cos\theta_{r}\sin\theta_{p}\sin\theta_{y}+\sin\theta_{r}\cos\theta_{y}\\ m_{31}=\sin\theta_{p}\\ m_{32}=-\sin\theta_{r}\cos\theta_{p}\\ m_{33}=\cos\theta_{r}\cos\theta_{p} \end{array}$$

where, $\theta_{p}$, $\theta_{r}$, and $\theta_{y}$ represent pitch, roll, and yaw angles of the aircraft, respectively. The four ground coordinates ([$T_{x},\;T_{y},\;T_{z}$]) can now be obtained by applying the rotation matrix and scale factor:

(6) $$\left[\begin{array}{c} T_{x}\\ T_{y}\\ T_{z} \end{array}\right]=s\left[\begin{array}{ccc} m_{11} & m_{21} & m_{31}\\ m_{12} & m_{22} & m_{32}\\ m_{13} & m_{23} & m_{33} \end{array}\right]\left[\begin{array}{c} O_{x}\\ O_{y}\\ O_{z} \end{array}\right]+\left[\begin{array}{c} P_{x}\\ P_{y}\\ P_{z} \end{array}\right]$$

where *s* represents a scale factor that varies depending on the flight altitude. If the GPS coordinates (*P_x_*, *P_y_*, *P_z_*) that correspond to the position of an image acquired are added additionally, then the ground coordinates ($T_{x},\;T_{y},\;T_{z}$) for the 4 corner points of the image can finally be obtained.

**Figure 6:**
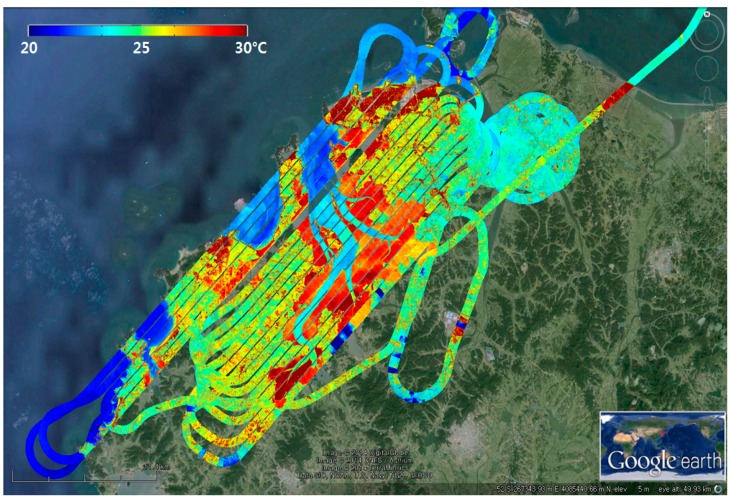
Geo-coded thermal infrared images overlaid on Google Earth image. The color scale represents apparent radiant temperature.

Although the accuracy of the ground coordinates obtained using this method depend on the accuracies of the GPS and IMU sensors used and may not be accurate enough, it is a very useful technique for the geometric correction of TIR images acquired over the ocean where there is almost no GCP available. Figure 6 is an example of geometric correction applied to a TIR image and overlaid on Google Earth. The temperatures presented in different colors represent the apparent radiant temperatures and not the kinetic temperatures of the objects themselves. It is observed that the water has a cooler radiant temperature than the land. Some land areas (e.g., tidal flat) have much higher radiant temperature than others because the tidal flats are usually composed of water-saturated bare soils and they seem to be rapidly heated by solar energy during ebb tide.

## 4. Coastal Monitoring Using the Airborne Sensors

### 4.1. Generation of Intertidal Flat DEM

An intertidal flat is the one of the difficult regions from which to extract topographic height. The direct measurement of topographic height in intertidal flats is usually restricted by tide (*i.e.*, the exposure time is too short) and muddy soils (*i.e.*, it is difficult to move around in the tidal flat). The waterline method for generating DEM has limitations because it requires numerous multi-temporal datasets acquired at different tide heights. Single-pass SAR interferometry was a common method to generate DEM without acquiring a multi-temporal dataset. Thus, an airborne SAR system can be used to measure the topographic height in the intertidal flats during low tide. We believe that the airborne SAR system further enables the monitoring of significant topographic changes caused by sediment transportation and tidal channel migration. In this study, dual-channel SAR data were acquired using the airborne SAR sensor over the west coast of the Korean peninsula. The basic radar parameters and specifications of the airborne SAR system during the data acquisitions are shown in Table 3. The first antenna of the SAR system transmitted radar signals and both antennas received the backscattered signals (bi-static mode). Both SAR antennas were mounted on the left side of the aircraft with a cross-track baseline. The relative positions of the IMU, GPS, and two SAR antennas were precisely surveyed using 3-D laser scanner and total station. This information was used for the motion compensation of SAR data as well as SAR interferometry calculations.

**Table 3:** Specification of airborne SAR sensor during XTI and ATI data acquisitions.

Parameter	Value (XTI)	Value (ATI)
Acquisition date	25 June 2013	17 September 2014
Pulse repetition frequency (PRF)	1000 Hz	1000 Hz
Baseline length	35.1 cm	37.8 cm
Baseline orientation angle	120°	-
Altitude	600 m	457 m
Velocity	41.62 m/s	45.99 m/s
Look angle (near/far)	30°/53°	17°/54°
Slant range (near/far)	696 m/1018 m	479 m/801 m
Range swath	322 m	322 m
Range resolution	0.314 m	0.314 m
Azimuth resolution	0.1 m	0.1 m

**Figure 7:**
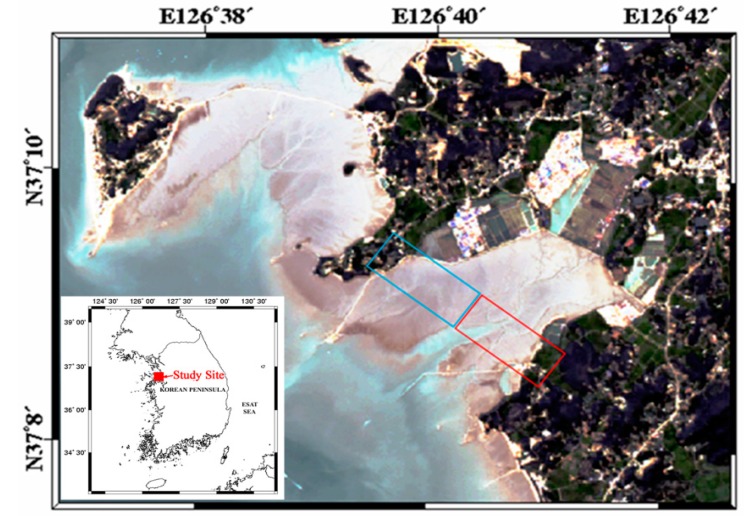
Study site for single-pass airborne SAR cross-track interferometry.

**Figure 8:**
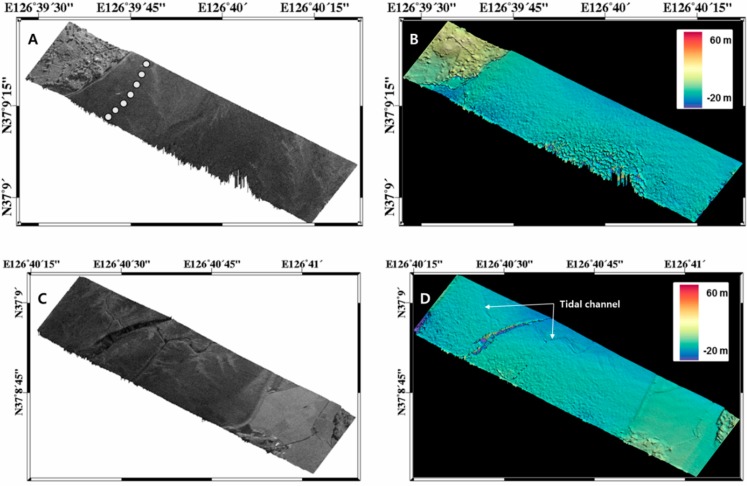
Generated topographic height over the intertidal flat shown in Figure 7.

**Figure 9:**
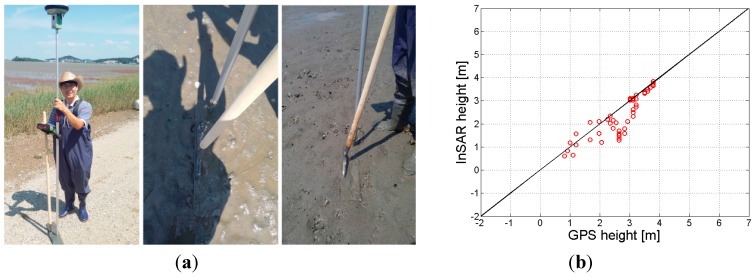
(**a**) GPS tracking along a transect line of intertidal flat in the study site; (**b**) Comparison between airborne InSAR derived height and the in-situ (GPS) measurements.

One of the study sites is an intertidal flat located near Jebu island in Hwaseong city (Figure 7). The site is characterized by a high tidal range (up to 9 m) and muddy soils. Because the topographic height variation over the intertidal flat was relatively small (<10 m), high sensitivity in height was required. For this, we collected single-pass interferometric data with a larger baseline of 35.1 cm and with low incidence angle ranges (less than 53°). The three dimensional position of a target ($\vec{T})\;$ including topographic height at each pixel can now be calculated using the following equations [11]:

(7) $$\vec{T}=\vec{P}+\rho \hat{\ell }$$

(8) $$\vec{T}=\vec{P}+\rho \left(\begin{array}{c} \frac{\lambda f}{2V}\hat{V}\\ +\frac{\frac{B^{2}}{2\rho }-\frac{\lambda \phi }{2\pi }\left(1+\frac{\lambda \phi }{4\pi \rho }\right)-\left(\vec{B}\cdot \hat{V}\right)\frac{\lambda f}{2V}}{B\sqrt{1-{\left(\hat{B}\;\cdot \;\hat{V}\right)}^{2}}}\frac{\left(\vec{V}\times \vec{B}\right)\times \vec{V}}{\left|\left(\vec{V}\times \vec{B}\right)\times \vec{V}\right|}\\ \pm \sqrt{1-{\left(\frac{\lambda f}{2V}\right)}^{2}-{\left(\frac{\frac{B^{2}}{2\rho }-\frac{\lambda \phi }{2\pi }\left(1+\frac{\lambda \phi }{4\pi \rho }\right)-\left(\vec{B}\;\cdot \;\hat{V}\right)\frac{\lambda f}{2V}}{B\sqrt{1-{\left(\hat{B}\;\cdot \;\hat{V}\right)}^{2}}}\right)}^{2}}\frac{\left(\vec{V}\times \vec{B}\right)}{\left|\left(\vec{V}\times \vec{B}\right)\right|} \end{array}\right)$$

where, $\vec{P}={\left(P_{x},\;P_{y},\;P_{z}\right)}^{t}\;$ is an GPS position of the first antenna (reference), ρ is the range distance between the target and the first antenna, $\hat{\ell }$ is an unit look vector that is pointing the target, and λ is the radar wavelength. Because a SAR data obtained from the first antenna provides the range (ρ) and Doppler frequency (f), an interferometric phase (ϕ) that is formed by multiplying the complex value of a pixel in one SAR data by the complex conjugate of the corresponding pixel in the second SAR data, can be used to determine the look vector accurately, with the information of baseline vector ($\vec{B})$ and velocity vector ($\vec{V}$). Figure 8 shows the generated topographic heights (DEM) for the study site. In these figures, we can observe typical intertidal topographic features, tiny channels, rocky dunes, and seaward gentle slopes. We conducted GPS tracking along a transect line (white dots in Figure 8A) and compared the airborne SAR derived heights with the GPS measurements. The comparison result showed a good agreement with the root-mean-square error (RMSE) of 0.54 m (Figure 9).

### 4.2. Measurement of Coastal Surface Current

**Figure 10:**
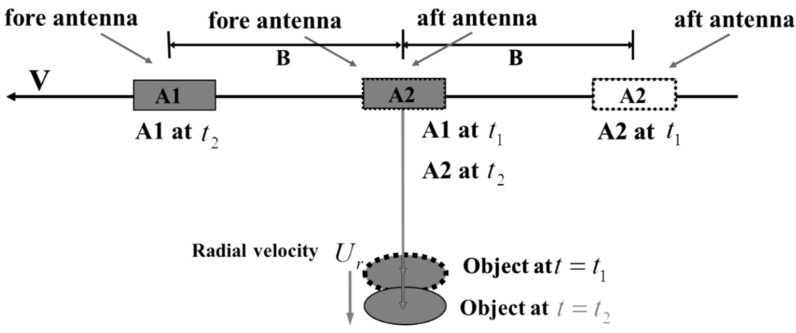
Geometry of along-track interferometric SAR (ATI).

The developed airborne system has the capability to measure and detect the velocity of the ocean (surface current) and moving targets such as vessels using two SAR antennas installed in the along-track direction (ATI). A moving target in radial motion causes a differential phase shift that can be measured by the interferometry technique. A moving target’s signals in SAR images are azimuthally displaced according to its radial velocity and superimposed at a displaced location upon non-moving targets. It is a slightly different story for surface current, where the entire distributed ocean surface causes the phase difference. Nonetheless, the surface velocity ($U_{h}$) of the ocean and moving targets can be detected by along-track interferometry (ϕ) using the following equations:

(9) $$U_{h}=-\frac{\lambda }{2\pi }\frac{V}{B}\frac{\phi }{sin\theta }$$

where B is an along-track baseline between two SAR antennas, V is a velocity of the aircraft, and θ is an incidence angle of the target (Figure 10).

**Figure 11:**
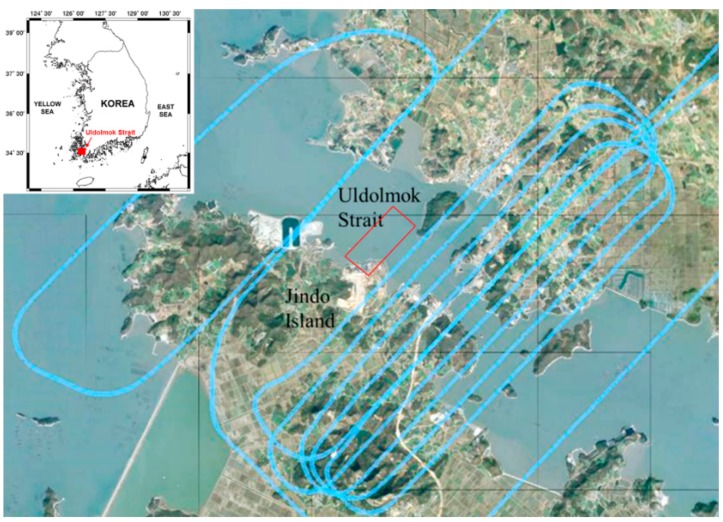
Airborne SAR ATI data acquisition near Uldolmok Strait on 17 September 2014. Flight tracks are shown as a blue line, and red rectangle represents the ATI SAR data coverage used in Figure 12.

**Figure 12:**
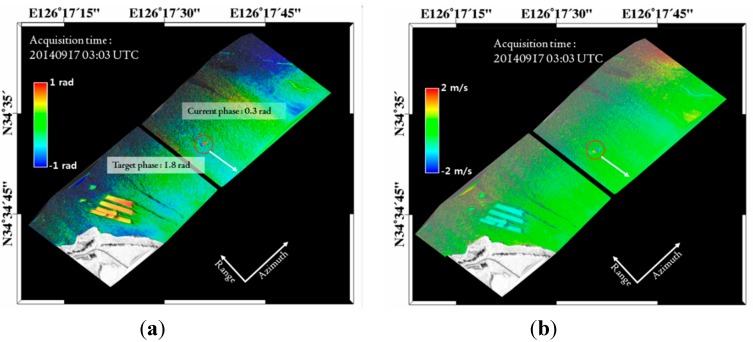
(**a**) Along-track interferogram of the acquired airborne SAR images containing a moving ship; (**b**) Velocity map calculated from the along-track interferogram.

**Figure 13:**
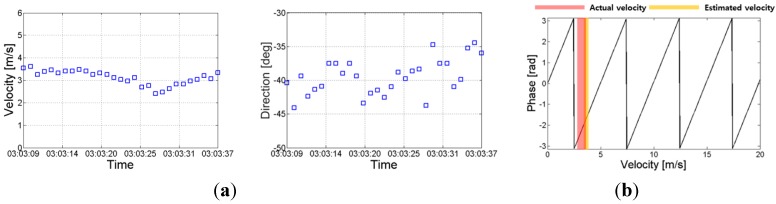
(**a**) GPS-measured ship velocities (speed and direction); (**b**) Estimated unwrapped ATI-velocity of a moving ship.

Based on this technique, the SAR data were acquired on 17 September 2014, at Uldolmok Strait by Jindo Island, in the southwest corner of South Korea (Figure 11). Uldolmok Strait is also known for its extremely high tidal-water speed, which exceeds 6.5 m/s with its narrow width at approximately 300 m. During the airborne ATI SAR data acquisition, a vessel with a corner reflector and GPS installed was operated simultaneously. The along track baseline of the airborne SAR antennas was 37.8 cm measured with Leica Geosystems Total Stations (Table 3). Figure 12a shows the resulting ATI phase calculated from two single look complex (SLC) data of both channels. The final surface velocity map (Figure 12b) can simply be obtained using the Equation (9) by considering local incidence angle. The velocity of the surface current varies from nearly zero to 1.0 m/s, which was much slower than expected. This is because the ATI data was obtained during the tidal phase change from ebb tide to flood tide. Although the absolute verification of the surface current was not possible owing to a lack of *in situ* data, the velocity of moving ship where a corner reflector was installed was compared with GPS measured velocities. The along-track interferometric phase of the moving ship was about 1.8 radian (which corresponds to the surface velocity of −1.4 m/s, where − indicates the target is receding from the radar), while the average speed extracted from GPS was about 3.4 m/s (Figure 13a). Although radial motion was considered, the estimated speed from the along-track interferometric SAR was significantly lower than the measured speed. This was caused by the relatively high speed of the ship so that the target phase exceeded the modulo of the 2π phase (Figures 13b). After considering the ambiguity 2nπ (*n = 1*), the difference was about 0.1 m/s.

### 4.3. High-Resolution Sea Surface Temperature in Coastal Area

**Figure 14:**
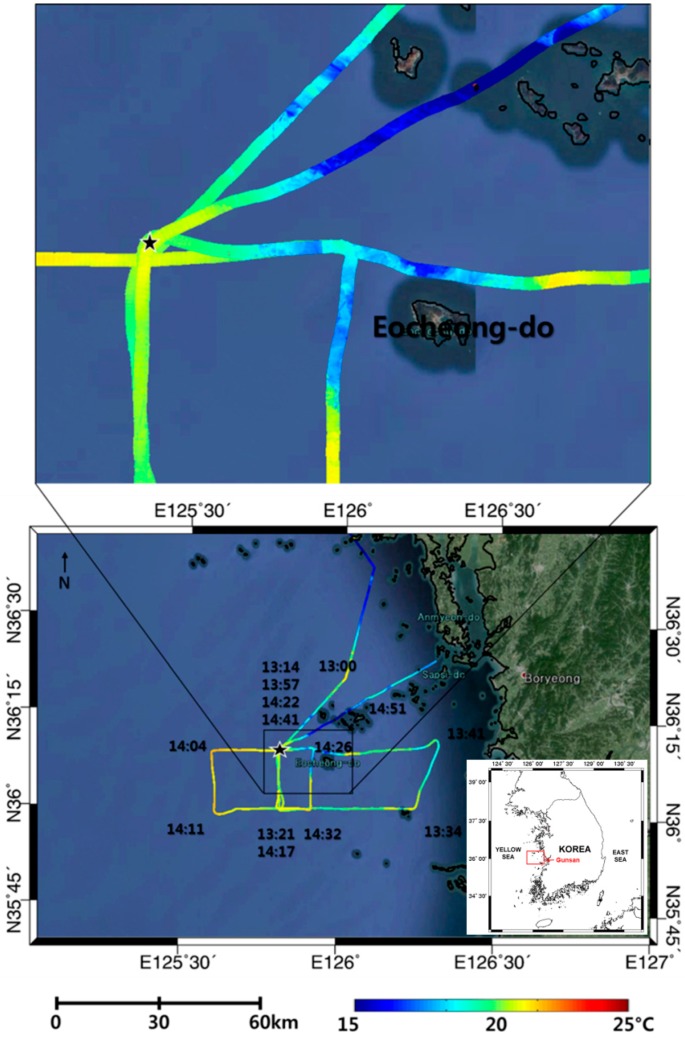
Airborne thermal infrared images overlaid on Google Earth (25 June 2013).

**Figure 15:**
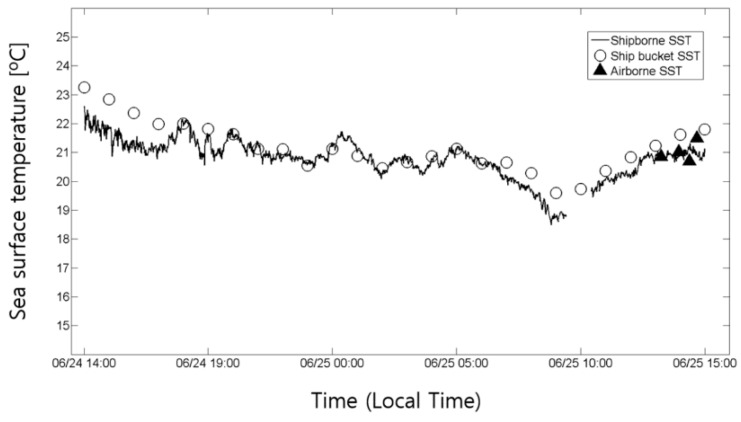
Comparison of variation in sea surface temperature for 25 h (24–25 June 2013).

The western coast of the Korean Peninsula is a semi-closed bay and has a large tidal range, and thus the area is very dynamic and vulnerable. It is essential to monitor the fine-scale structures of oceanic features in this area. In this study, a cost-effective airborne thermal infrared sensor is considered to verify its capability of extracting sea surface temperature in high-resolution (0.6 m pixel resolution at 500 m above sea level) and to monitor changes in the coastal environment. The swath of airborne thermal infrared images is narrow with 414 m at 500 m above sea level, but with long strips we can observe the fine-scale structures such as the oceanic front, foam, and upwelling features within thermal infrared images. A total of 18 airborne acquisitions were carried out along the western coast of the Korean Peninsula during the period between 23 May 2012 and 27 November 2014. One of the examples is shown in Figure 6. In particular, simultaneous observations of airborne and ship-borne TIR sensors were carried out on 25 June 2013 (Figure 14). On the same date, in-situ sea surface temperatures were also obtained from the measurement of sea surface water sampled by bucket at every hours and continued for 25 h (24–25 June 2013), mooring off the fixed point (★ in Figure 14) near the Eocheong-do in Gunsan city. The airborne sea surface temperatures acquired on 25 June, 2013, were compared with ship-borne thermal infrared data and *in situ* data (Figure 15). Four airborne thermal infrared data were acquired over the fixed point during the in-situ measurements. The comparison result showed reasonable temporal trends between the ship-borne SST and the in-situ SST, with the root-mean-square error (RMSE) of 0.6 °C. The RMSE of airborne SST was about 0.4 °C when they were compared with in-situ SST. Because the airborne TIR sensor can provide very high-resolution SST data (~0.6 m), small-scale oceanic features were easily detected. The relatively low temperature band near Eocheong-do, which has never been reported from space-borne SST data, was well observed and revealed as upwelling signatures caused by bottom topography and flooding tide (Figure 14).

### 4.4. Monitoring of Submarine Groundwater Discharge (SGD)

Submarine groundwater discharge (SGD) is defined broadly as any and all flow of water across the seabed from land to sea, and includes several components of subsurface flow including terrestrial freshwater and recirculated seawater [12,13]. SGD has recently been recognized as a ubiquitous phenomenon that can strongly influence the coastal water and drive ecosystem changes [13,14,15]. Little research has been conducted for detecting the location of SGD using thermal infrared sensors. In this study, the airborne TIR and SAR sensors were used to improve the detectability of SGD locations.

**Figure 16:**
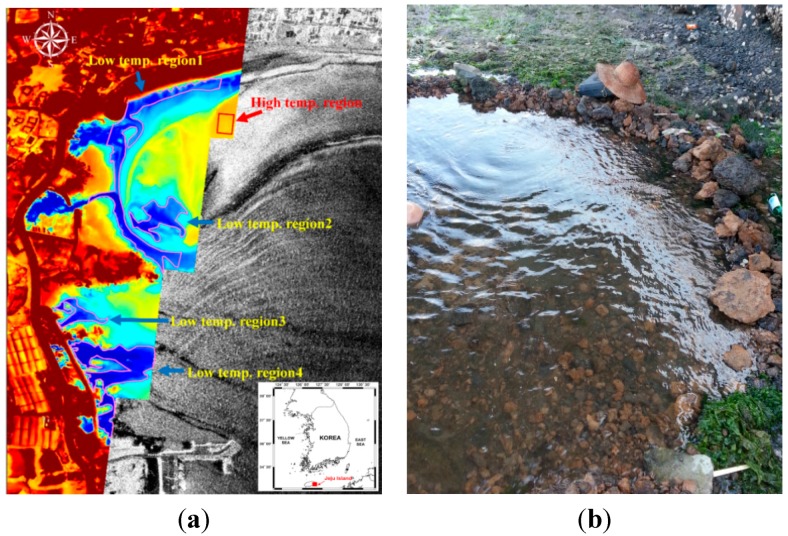
(**a**) Airborne TIR and SAR images that were acquired simultaneously at Jeju island on 21 August 2013, and (**b**) a photo showing a groundwater discharge in the area.

The study site is the eastern part of Jeju Island, which is located at the southern part of South Korea (Figure 16). To combine information from the two different sensors, co-registration between TIR and SAR images was performed. Then the regions of interest (ROIs) showing low or high temperatures from the TIR images were created. The areas showing relatively low radar backscattering from the SAR image among ROIs were selected as the most probable SGD locations (Figure 16a). We were actually able to find SGD in these locations (Figure 16b).

## 5. Conclusions

Coastal monitoring with an airborne remote sensing system is highly required to respond to changes in environment caused by climate change and human impact. In this study, an airborne remote sensing system with onboard SAR and TIR sensors was implemented, and several algorithms suited for coastal monitoring were also developed. The airborne surveys were carried out in the west and south coast of the Korean Peninsula and coastal images were obtained successfully. The topographic heights in a gentle intertidal flat, surface currents in a narrow strait, sea surface temperatures around coastal islands, and potential locations of submarine groundwater discharge were all effectively extracted and detected from the airborne remote sensing system, and believed to be useful for coastal monitoring.

In contrast with sun-synchronous orbiting satellites (which pass over the same part of the earth at roughly the same local time), the airborne system can collect coastal images during low tide or high tide depending on the environmental parameters. This enables us to monitor any subtle changes in the coastal area quickly, and it also makes possible the detection of emergent coastal disasters such as oil spills and red tides. Because of these advantages of the airborne remote sensing system in coastal monitoring, not only the above-mentioned parameters but also more applications such as oyster migration mapping in tidal flats and sediment transportations will be developed.

## References

[1] Kim D.-J., Moon W.M., Kim Y.S. (2010). Application of TerraSAR-X data for emergent oil-spill monitoring. IEEE Trans. Geosci. Remote Sens..

[2] Kara A.B., Wallcraft A.J., Hurlburt H.E. (2005). Sea surface temperature sensitivity to water turbidity from simulations of turbid black sea using HYCOM. J. Phys. Oceanogr..

[3] Fu L.L., Holt B. (1982). Seasat views oceans and sea ice with synthetic aperture radar. NASA Tech. Rep..

[4] Chapin E., Hensley S., Michel T.R. Calibration of an across track interferometric P-band SAR. Proceedings of the 2001 IEEE Geoscience and Remote Sensing Symposium.

[5] Rosen P.A., Hensley S., Joughin I.R., Li F.K., Madsen S.N., Rodriguez E., Goldstein R.M. (2000). Synthetic aperture radar interferometry. Proc. IEEE..

[6] Chandrasekhar S. (1960). Radiative Transfer.

[7] Price J.C. (1983). Estimating surface temperatures from satellite thermal infrared data—A simple formulation for the atmospheric effect. Remote Sens. Environ..

[8] Ricchiazzi P., Yang S., Gautier C., Sowle D. (1998). SBDART: A research and teaching software tool for plane-parallel radiative transfer in the earth’s atmosphere. Bull. Am. Meteorol. Soc..

[9] Kim D.-J., Cho Y., Kang K., Kim J., Kim S. (2013). Development of Airborne Remote Sensing System for Monitoring Marine Meteorology (Sea Surface Wind and Temperature). Sea.

[10] Wolf P.R., Dewitt B.A. (2000). Elements of Photogrammetry with Applications in GIS.

[11] Hensley S. A combined methodology for SAR interferometric and stereometric error modeling. Proceedings of the 2009 IEEE Radar Conference.

[12] Moore W.S. (1999). The subterranean-estuary: A reaction zone of groundwater and sea water. Mar. Chem..

[13] Burnett W.C., Bokuniewicz H., Huettel M., Moore W.S., Taniguchi M. (2003). Groundwater and pore water inputs to the coastal zone. Biogeochemistry.

[14] D’Elia C.F., Webb K.L., Porter J.W. (1981). Nitrate-rich groundwater inputs to Discovery Bay, Jamaica: A significant source of N to local coral reefs?. Bull. Mar. Sci..

[15] Valiela I., Costa J., Foreman K., Teal J.M., Howes B.L., Aubrey D.G. (1990). Transport of groundwater-borne nutrients from watersheds and their effects on coastal waters. Biogeochemistry.

